# Research trends and knowledge structure of pancreatic cancer and type 2 diabetes mellitus research: a two-decade CiteSpace analysis

**DOI:** 10.3389/fendo.2026.1945735

**Published:** 2026-09-30

**Authors:** Zilin Dong, Tingting Zhou, Shengwei Ma, Xuannan Chen, Yu Lai

**Affiliations:** 1 Acupuncture and Tuina School, Chengdu University of Traditional Chinese Medicine, Chengdu, China; 2 School of Clinical Medicine, Chengdu University of Traditional Chinese Medicine, Chengdu, China; 3 School of Basic Medicine, Chengdu University of Traditional Chinese Medicine, Chengdu, China

**Keywords:** bibliometric analysis, pancreatic cancer, type 2 diabetes mellitus, insulin resistance, new-onset diabetes

## Abstract

**Background:**

Pancreatic cancer and type 2 diabetes mellitus (T2DM) are closely linked through complex epidemiological and biological interactions. However, the development, knowledge structure, and research trends of this field have not been systematically characterized. This study aimed to investigate the research landscape and knowledge structure of pancreatic cancer and T2DM research from 2006 to 2025.

**Methods:**

We conducted the primary bibliometric analysis using WOSCC records, and separately analyzed PubMed-indexed clinical trials as a supplementary dataset. A total of 1,989 WOSCC publications were included in the bibliometric analysis, while 54 PubMed-indexed Clinical Trial records were included in the supplementary clinical trial assessment. CiteSpace was used to perform collaboration, co-citation, keyword, timeline, and burst analyses of the WOSCC dataset.

**Results:**

The number of publications increased steadily over the study period, particularly after 2015. The United States and China were the leading contributors, while the University of California System and Harvard University were among the most productive institutions. Major research themes included cancer risk, insulin resistance, metabolic inflammation, incretin-based therapies, and new-onset diabetes. Keyword evolution and burst analyses indicated increasing attention to mechanistic investigation, biomarker discovery, risk stratification, and precision medicine approaches.

**Conclusion:**

This study characterizes the development and knowledge structure of research on the relationship between pancreatic cancer and T2DM and highlights major research themes, emerging hotspots, and future directions for mechanistic investigation and clinical translation.

## Introduction

Pancreatic cancer is one of the most lethal malignancies worldwide, with pancreatic ductal adenocarcinoma (PDAC) accounting for the vast majority of cases. Therefore, in research and clinical practice, the term “pancreatic cancer” usually refers to PDAC. Globally, the lifetime risk of pancreatic cancer is about 0.89%, and the risk varies greatly in different regions, ranging from about 0.15% in Central Africa to 2.06% in Western Europe. At the same time, the lifetime risk of death due to pancreatic cancer is also high, about 0.85% (1). Pancreatic cancer is influenced by both genetic and lifestyle factors, including smoking, alcohol consumption, diet, and obesity (2). Accumulating evidence suggests that type 2 diabetes mellitus (T2DM) is an important risk factor for pancreatic cancer. T2DM is associated with several pancreatic alterations, including islet amyloid deposition (3), reduced beta cell mass (4), and increased pancreatic fat (5). These alterations may create a pancreatic microenvironment characterized by chronic inflammation and cellular stress. This direct pancreatic involvement provides a convincing biological basis for exploring the potential role of T2DM in the pathogenesis of pancreatic cancer. Therefore, studying the relationship between pancreatic cancer and T2DM has become a growing research domain.

T2DM is a complex metabolic disorder characterized by insulin resistance and progressive dysfunction of pancreatic β cells. It is driven by both genetic predisposition and lifestyle factors (6, 7). A prospective study of over 500,000 Chinese adults and a meta-analysis of 22 cohort studies demonstrated that T2DM increases the risk of pancreatic cancer by approximately 52% to 87%, and that even before blood glucose levels reach the diagnostic threshold for diabetes, higher glycemic levels are progressively associated with an increased incidence of pancreatic cancer (8). Moreover, T2DM may remodel the pancreatic tumor microenvironment by expanding tumor-promoting myofibroblastic pancreatic stellate cells and reducing CXCL13^+^ tumor immunity- and angiogenesis-promoting Pafs, which diminishes anti-tumor immune responses and vascularization (9). These studies have revealed that systemic metabolic disorder and local cell remodeling jointly promote the occurrence of pancreatic cancer. Beyond these, T2DM may promote pancreatic cancer through hyperinsulinemia, which directly stimulates acinar cells via insulin receptors, increasing digestive enzyme production, local inflammation, and acinar-to-ductal metaplasia, thereby providing mechanistic evidence for facilitating tumor initiation (10). PDAC itself can induce diabetes, often referred to as pancreatogenic or type 3c diabetes (11). These findings highlight the complex interplay between pancreatic cancer and T2DM. To further investigate the link, it is necessary to comprehensively and systematically evaluate the existing research.

Bibliometrics is an interdisciplinary field dedicated to the systematic analysis of published studies (12). It conducts statistical research on published literature and its related metadata, such as abstracts, keywords, and citations, to analyze patterns, quantify impacts, and thereby reveal relationships between academic works (13). Citespace is a powerful visualization software developed by Professor Chaomei Chen for analyzing the evolution of research fields (14). Using citation and network analysis, we map the evolution of research on pancreatic cancer and T2DM. While studies have moved from epidemiology to mechanisms and treatment, systematic bibliometric analyses of their complex interaction are scarce. This study uses CiteSpace to visualize key research areas, intellectual structure, and emerging trends, providing insights for etiology and therapy.

## Methods

### Data collection and processing

The Web of Science Core Collection (WOSCC) served as the primary dataset for bibliometric analyses, while PubMed was queried separately to characterize clinical trial research on pancreatic cancer and T2DM and was used exclusively for a supplementary descriptive analysis of clinical research trends. WOSCC was selected as the primary database for citation-based visualization analysis because of its comprehensive citation records and compatibility with CiteSpace (15). Relevant publications were retrieved from the Science Citation Index Expanded (SCIE) database within WOSCC. The literature search was conducted using the advanced search function of WOSCC, following standardized field tag conventions.

For the WOSCC dataset, Topic Search (TS) was conducted using pancreatic cancer-related terms (“pancreatic cancer”, “pancreatic neoplasm*”, “pancreatic carcinoma*”, “pancreatic tumor*”, “pancreatic malignanc*”, and “adenocarcinoma of the pancreas”) combined with T2DM-related terms (“type 2 diabetes mellitus”, “T2DM”, “T2D”, and related synonyms). For the supplementary PubMed Clinical Trial dataset, both MeSH terms and title/abstract keywords were used, including “Pancreatic Neoplasms” [MeSH] and “Diabetes Mellitus, Type 2” [MeSH]. The complete search strategy is presented in **Figure 1** and detailed in the Data availability statement.

**Figure 1:**
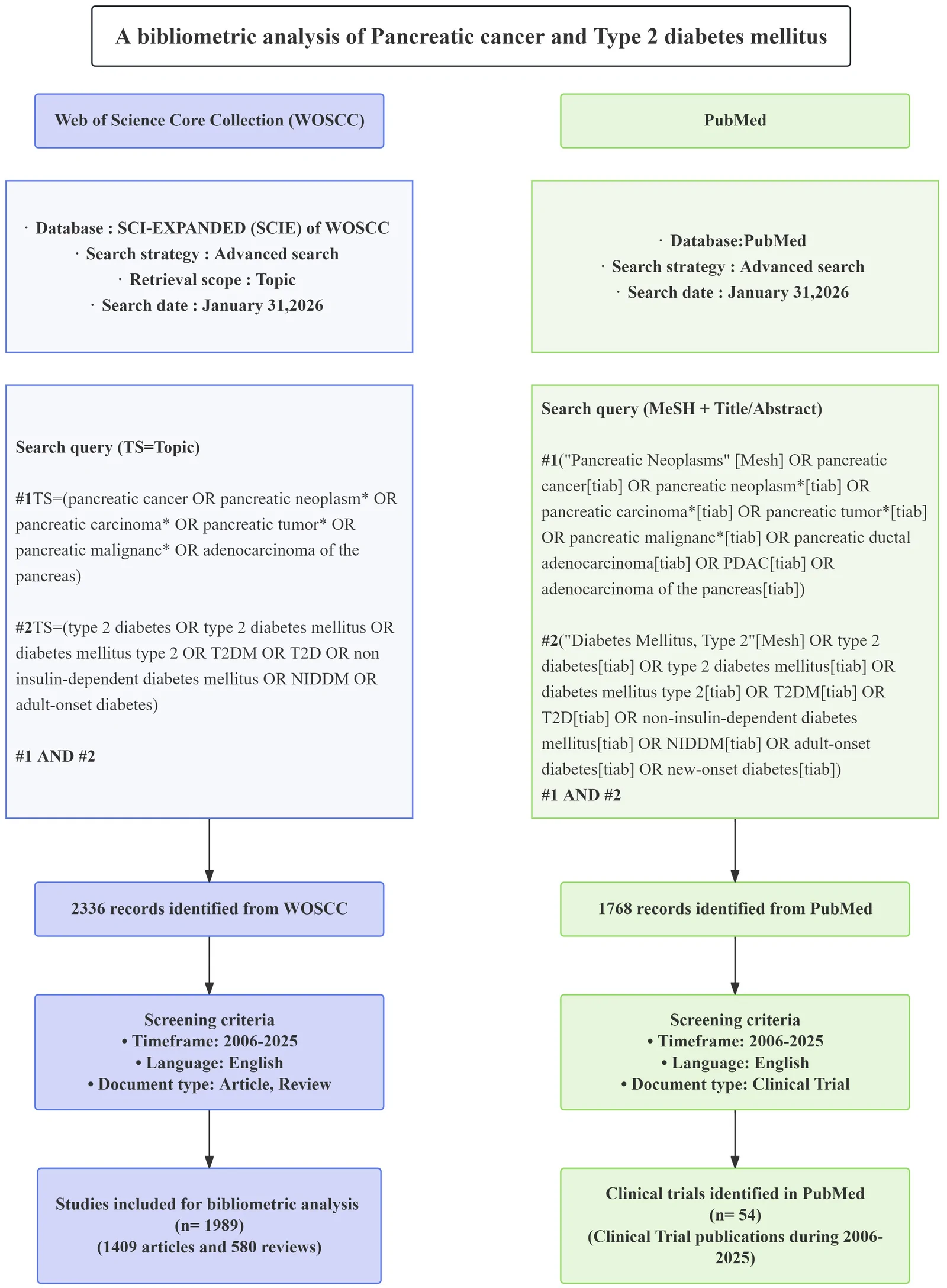
Retrieval process. This image presents a flowchart illustrating the literature retrieval method for our investigation.

The inclusion criteria were as follows: (1) For the WOSCC dataset, only articles and reviews published in English were included. For the PubMed dataset, records indexed as Clinical Trial were retrieved to characterize clinical research trends; (2) Publications indexed between January 1, 2006, and December 31, 2025; and (3) Studies focusing on pancreatic cancer and T2DM or their biological, epidemiological, diagnostic, or therapeutic interactions. (4) For the WOSCC dataset, duplicate records were identified and removed using the built-in “Remove Duplicates” function in CiteSpace before bibliometric analysis. Because the PubMed clinical trial dataset was analyzed separately and not merged with the WOSCC dataset, cross-database deduplication was not applicable. All eligible records were exported in plain text format with “full records and cited references” selected. Records were downloaded in batches of 500 and stored as separate text files (e.g., “download_1–500.txt”, “download_501–1000.txt”). To ensure data stability and minimize potential bias caused by database updates, all records were retrieved on January 31, 2026. Due to indexing delays, publications from 2025 may be slightly incomplete. Two researchers independently screened titles and abstracts for eligibility. Records were excluded mainly due to irrelevance to pancreatic cancer or T2DM, non-eligible publication types, or failure to meet the predefined inclusion criteria. We resolved disagreements about study inclusion through discussion until consensus was reached. For the WOSCC dataset. After WOSCC data cleaning, 1,989 valid publications were retained for subsequent analysis. The PubMed clinical trial dataset was analyzed separately and was not included in the CiteSpace dataset. Prior to analysis, bibliographic records were manually checked to reduce inconsistencies in author and institutional names. Obvious variations in author names (e.g., abbreviated versus full-name forms) were standardized when they clearly referred to the same individual. Institutional names were standardized at the parent-organization level, and affiliated departments, hospitals, and research centers belonging to the same institution were merged when appropriate. National research institutes and international organizations were treated as independent entities.

### Bibliometrics and visualization analysis

CiteSpace (Version 6.4.R1) was used for all bibliometric analyses. The following settings were applied: time span = 2006-2025; years per slice = 1; node selection = g-index (k = 25); term sources = Title, Abstract, Author Keywords, and Keywords Plus; No additional pruning procedure was applied unless specified. Link retaining options followed the default CiteSpace settings. Network analyses were performed separately for countries, institutions, authors, cited references, cited journals, and keywords. Unless otherwise specified, default CiteSpace parameters were retained. The knowledge map generated by CiteSpace consists of nodes and links. Nodes represent analytical units such as countries, institutions, authors, keywords, and references, while links represent relationships including collaboration, co-occurrence, or co-citation. Node size is directly proportional to its frequency of appearance or citations, and node color reflects temporal information, visualizing the dynamic evolution of the research field. Betweenness centrality is used as a key structural indicator to evaluate the importance of nodes in a network, which is a measure derived from network theory that quantifies the degree to which a node is located on the shortest path between other nodes, defined as:


$$g\left(v\right)=\sum_{s\neq v\neq t}\frac{\sigma_{st}\left(v\right)}{\sigma_{st}}$$

Among them, σ_st (v) represents the number of shortest paths between a node pair (*s*, ^t^) that passes through node *v*, and σ_st represents the total number of shortest paths between ^s^ and *t* (16). Nodes with betweenness centrality ≥ 0.1 are generally considered key connections in the network, which may indicate influential publications or key turning points in the development of research fields (17). Burst detection was applied to identify references and keywords that exhibited sudden increases in frequency within a specific time period. This time series–based method is widely used to detect emerging research fronts and shifts in scholarly attention (18). The g-index parameter in CiteSpace is controlled by the k value and is used to control node selection by integrating citation frequency with network structure. The g-index indicates that the top g publications collectively receive at least g² citations, meeting the following condition:


$$g^{2}\leq \text{k}\sum_{i\leq \text{g}}c_{i},k\in Z^{+}$$

where *ci* denotes the citation count of the i -th ranked article. In this study, a k-value of 25 was applied to the g-index for node selection. These indicators enabled the visualization and characterization of research productivity, collaboration patterns, and emerging themes within the literature.

## Results

### Analysis of annual publication volume

Variations in annual publication output provide a quantitative indicator for research activities and knowledge accumulation in specific fields. By analyzing changes in the number of publications over time, the field’s historical development can be studied and potential growth and expansion stages identified. **Figure 2** illustrates the number of annual publications related to the study of the relationship between pancreatic cancer and T2DM from 2006 to 2025. The X-axis is the year of publication, and the Y-axis is the number of publications. Overall, the annual publication volume exhibited a fluctuating yet steadily increasing trend throughout the study period, indicating sustained academic attention to this topic. The earliest article mentioned the roles of islet transplantation and VEGF-mediated angiogenesis in regulating glucose homeostasis (19), indicating that early studies focused on pancreatic vascular biology and β-cell-related diabetes research.

**Figure 2:**
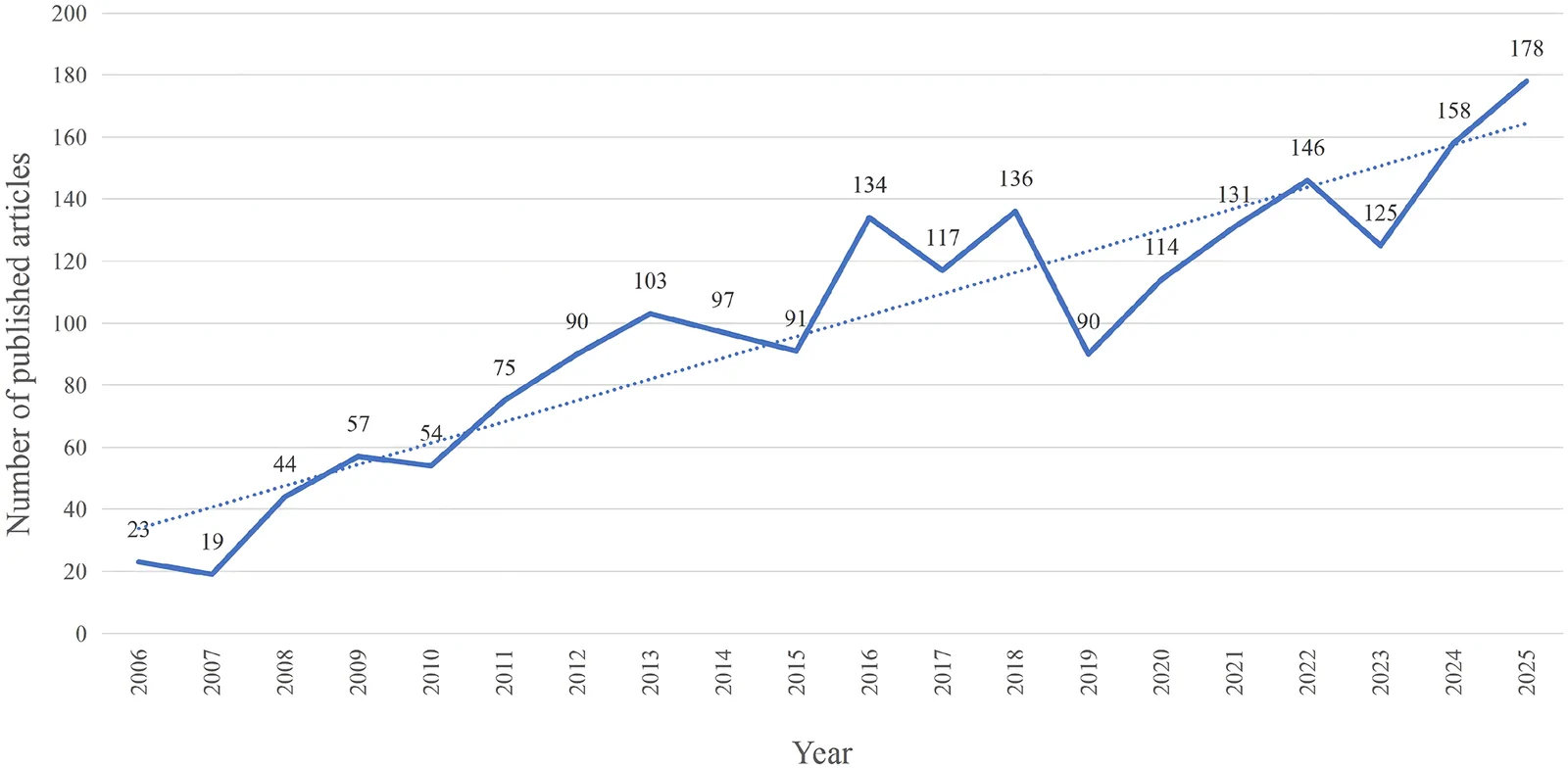
Trend chart of the annual publication volume (2006–2025).

Before 2010, research on the relationship between pancreatic cancer and T2DM was limited, and the number of publications per year was low. During this period, research mainly focused on basic clinical observations and epidemiological associations. Since 2011, the number of publications has increased steadily, reflecting the growing interest of the academic community in the two-way relationship between pancreatic cancer and T2DM. This growth became more apparent after 2015, indicating that research topics and study designs in this field are expanding. From 2016 to 2018, the average number of publications was 129, a significant increase from previous years. From 2021, the number of articles has remained consistently high, indicating the continued attention and maturity of this research field.

### Analysis of countries and institutions

**Table 1** summarizes the publications of the top 10 countries and institutions that contributed the most to research on the relationship between pancreatic cancer and T2DM. At the national level, the United States has the most publications (587 articles), followed by China (418) and the United Kingdom (175). These countries together account for the majority of the total research output in this field. The national cooperation network consists of 105 nodes and 832 links, reflecting extensive international cooperation. Nodes surrounded by purple rings indicate betweenness centrality values greater than 0.1, representing countries that serve as important connectors in the global cooperative network. As shown in **Figure 3A**, the United States and the United Kingdom exhibit high betweenness centrality, highlighting their mediating role in promoting international research cooperation. At the institutional level, the University of California System ranked first with 61 publications, followed by Harvard University (58) and the National Institutes of Health (50). The institutional collaboration network consists of 494 nodes and 2,011 links. As illustrated in **Figure 3B**, several leading institutions occupy central positions in the network, forming dense cooperative relationships with multiple partners, indicating a high level of institutional connections and knowledge exchange in this field.

**Table 1:** Top 10 countries and institutions with published articles.

Rank	Count	Country	Count	Institutions
1	587	USA	61	University of California System
2	418	China	58	Harvard University
3	175	United Kingdom	50	National Institutes of Health (NIH) - USA
4	153	Italy	47	University of Texas System
5	119	Japan	38	Institut National de la Sante et de la Recherche Medicale (Inserm)
6	106	Germany	38	Mayo Clinic
7	91	India	36	University of London
8	82	Canada	32	Karolinska Institutet
9	73	South Korea	31	Imperial College London
10	72	France	31	University of California Los Angeles

**Figure 3:**
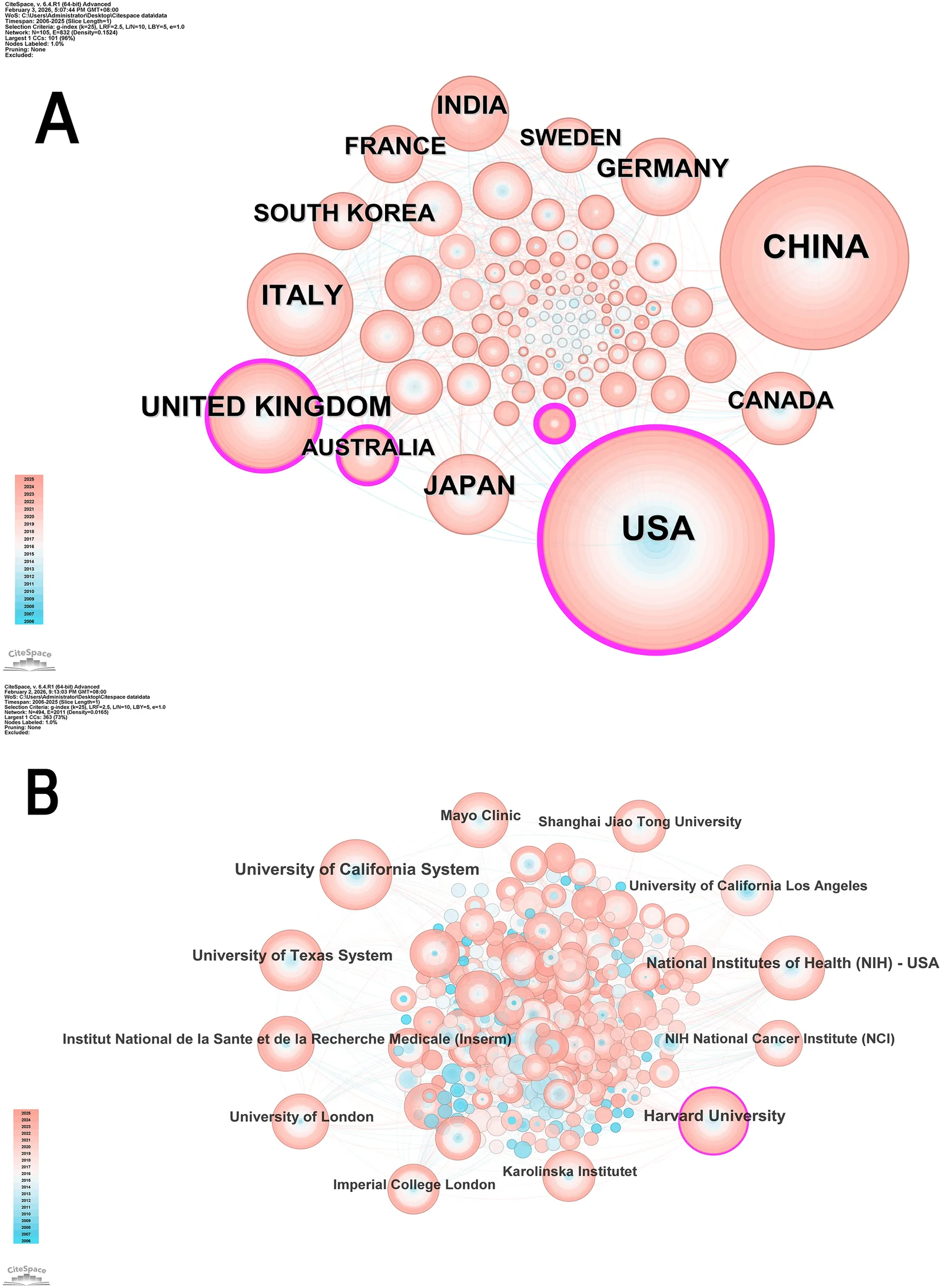
**(A)** Map of countries. **(B)** Map of institutions. The color gradient in the bottom-left corner indicates publication year, increasing from bottom to top. The size of each circle indicates the publication output of a country or institution. Lines connecting the circles represent international collaboration between countries or institutions.

### Analysis of authors and co-cited authors

**Table 2** summarizes the top 10 authors with the highest publication volume and the top 10 authors with the most co-citations. The top 10 prolific authors have published 61 articles, accounting for 4.95% of the total literature. Dana K. Andersen and Phil A. Hart are the authors with the highest output, each publishing 10 papers (0.81%). As shown in **Figure 4A**, the co-authorship network comprises 822 author nodes and 1,477 collaboration links, suggesting a relatively dispersed authorship structure. In terms of co-citation frequency, Donghui Li ranked first, with 198 publications citing one or more of the author’s works, reflecting substantial scholarly influence. The network of co-cited authors (**Figure 4B**) includes 964 nodes and 5,408 links, suggesting strong connectivity within the knowledge base. The high co-citation frequency of authors such as E. Giovannucci, R. Huxley, and Donghui Li indicates their central position.

**Table 2:** Top 10 authors and cocited authors.

Rank	Count	Authors	Count	Cocited authors
1	10	Dana K. Andersen	198	Donghui Li
2	10	Phil A. Hart	191	E. Giovannucci
3	7	Maxim S. Petrov	174	R. Huxley
4	6	Mark O. Goodarzi	171	A. E. Butler
5	5	Daniela Basso	163	R. Pannala
6	5	Suresh T. Chari	160	S. T. Chari
7	5	Guido Eibl	157	S. C. Larsson
8	5	Lijuan Zhang	149	Q. W. Ben
9	4	Habibul Ahsan	127	Amer. Diabetassoc.
10	4	Demetrius Albanes	109	M. Elashoff

**Figure 4:**
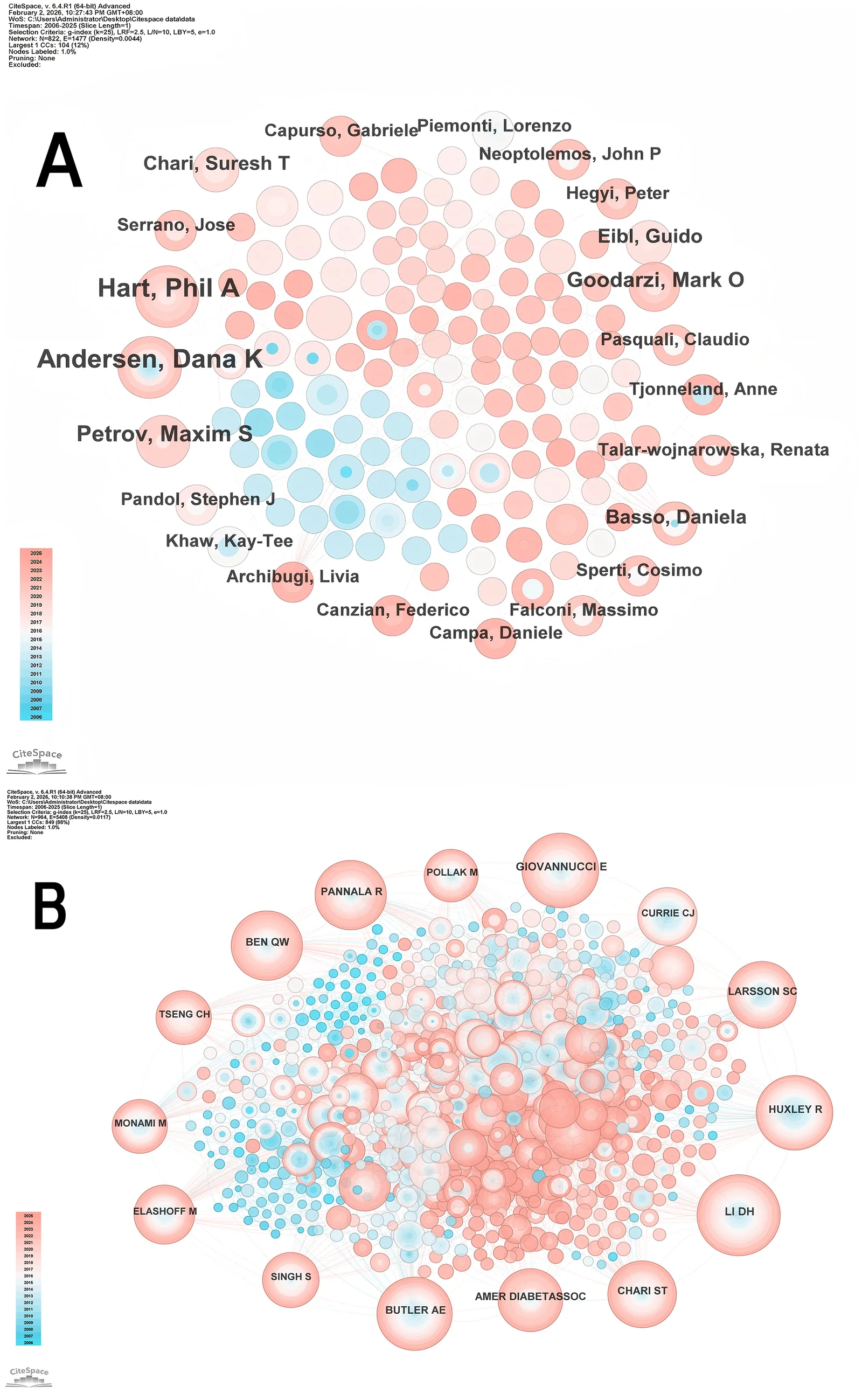
**(A)** Map of co-authors. **(B)** Map of co-cited authors. The coauthor network illustrates active collaboration patterns among researchers in the field. Each node represents an author, with node size proportional to publication output and link thickness indicating the strength of collaboration. In the co-cited network, nodes represent frequently cited authors, and their proximity reflects the degree of intellectual relatedness.

### Analysis of journals: overlay map

The dual-map overlay of journals displays results from the disciplinary distribution and citation trajectory visualizations at the journal level. As shown in **Figure 5**, citing journals are displayed on the left, while cited journals are shown on the right, with curved paths representing citation relationships between disciplines. The results indicate that articles published in journals in the Medicine, Medical, Clinical and Molecular Biology, and Immunology categories mainly cited journals in the domains of Molecular Biology, Genetics and Health, Nursing, and Medicine. Elliptical nodes represent the relative distribution of authors and publications, with the horizontal axis representing the number of authors and the vertical axis the number of publications. These citation trajectories suggest that research on pancreatic cancer and T2DM is mainly based in clinical medicine and molecular biomedical sciences, with a strong interdisciplinary knowledge flow between basic and clinical research.

**Figure 5:**
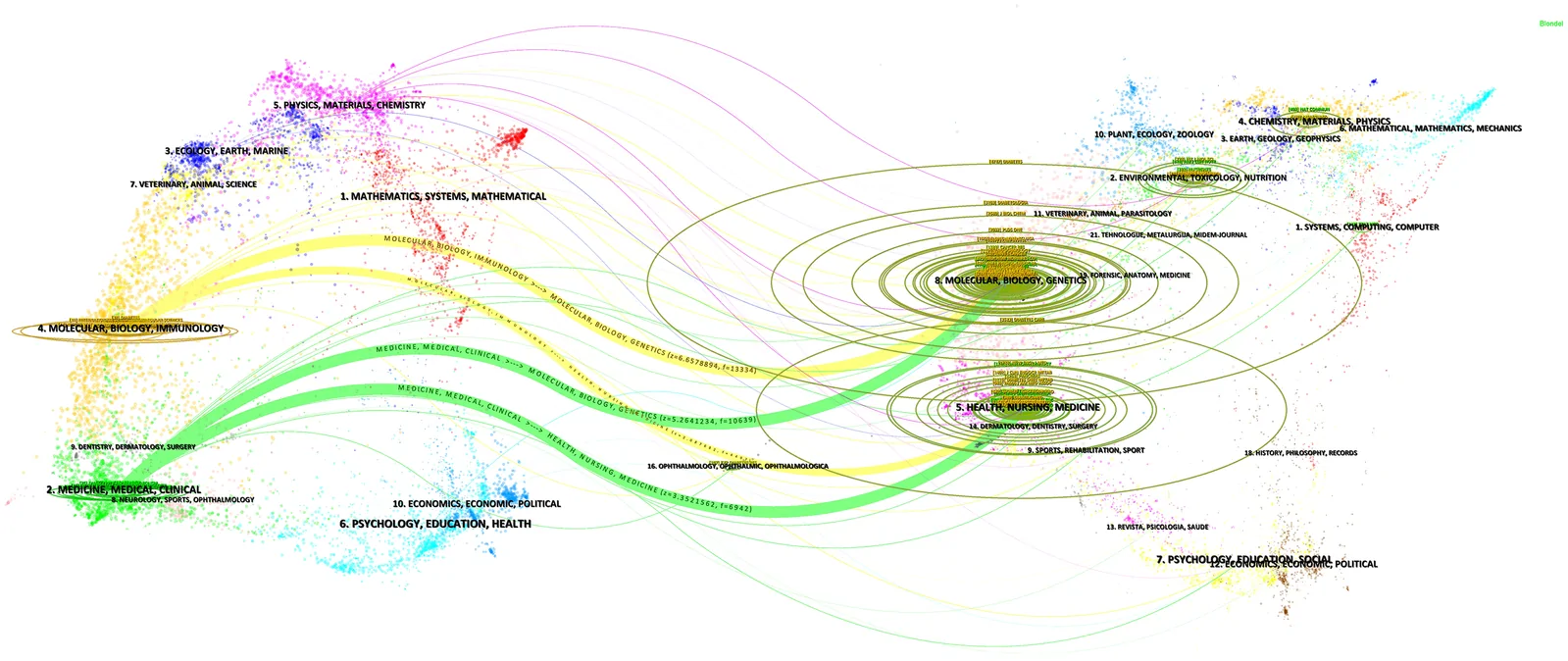
Overlay map of journals. The diagram depicts citation relationships within the relevant research domain.

### Analysis of reference

#### Co-cited representative reference analysis

Citation frequency is a key indicator for evaluating the contribution and influence of a publication in the research field (18). **Table 3** summarizes the 10 most frequently cited references on the relationship between pancreatic cancer and T2DM. Among these references, the most cited ones primarily focus on the epidemiological association between pancreatic cancer and diabetes, the impact of antidiabetic therapies on cancer risk, and the clinical significance of new-onset diabetes (NOD). Highly cited articles by Ben et al. and Giovannucci et al. systematically assess the risk relationship between pancreatic cancer and diabetes, which established diabetes as an important risk factor for pancreatic cancer (23, 24). Other influential studies, such as those by Currie et al. and Donghui Li et al., have investigated the impact of glucose adjuvant therapy and diabetes status on pancreatic cancer risk, emphasizing the complex interactions among metabolic disorders, treatment strategies, and cancer development (21, 22).

**Table 3:** The top 10 co-cited representative studies.

Rank	Cited frequency	Title	Journal	Reference
1	54	Pancreatitis, pancreatic, and thyroid cancer with glucagon-like peptide-1-based therapies	*Gastroenterology*	(20)
2	51	The influence of glucose-lowering therapies on cancer risk in type 2 diabetes	*Diabetologia*	(21)
3	49	Antidiabetic Therapies Affect Risk of Pancreatic Cancer	*Gastroenterology*	(22)
4	47	Diabetes mellitus and risk of pancreatic cancer: A meta-analysis of cohort studies	*European Journal of Cancer*	(23)
5	42	Diabetes, Pancreatogenic Diabetes, and Pancreatic Cancer	*Diabetes*	(11)
6	41	Diabetes and Cancer A consensus report	*Diabetes Care*	(24)
7	41	New Users of Metformin Are at Low Risk of Incident Cancer A cohort study among people with type 2 diabetes	*Diabetes Care*	(25)
8	38	Global cancer statistics 2020: GLOBOCAN estimates of incidence and mortality worldwide for 36 cancers in 185 countries	*CA-A Cancer Journal for Clinicians*	(26)
9	33	Marked Expansion of Exocrine and Endocrine Pancreas, With Incretin Therapy in Humans, With Increased Exocrine Pancreas Dysplasia and the Potential for Glucagon-Producing Neuroendocrine Tumors	*Diabetes*	(27)
10	32	Saxagliptin and Cardiovascular Outcomes in Patients with Type 2 Diabetes Mellitus	*New England Journal of Medicine*	(28)

In addition, multiple highly co-cited references elaborated on the bidirectional relationship between pancreatic cancer and diabetes. Andersen et al. provided an integrative clinical perspective, emphasizing that NOD may be an early manifestation of pancreatic cancer, not just a traditional risk factor (11). The research by Elashoff et al. and Butler et al. further examined pancreatic alterations and safety issues associated with incretin-based therapies, prompting extensive discussion of their potential association with pancreatic diseases (20, 27). Several highly cited studies were published in high-impact journals such as *Gastroenterology*, *Diabetes Care*, and *Diabetes*, which underscores the authority of these journals in shaping research directions in this field.

#### Co-cited references cluster analysis

A co-citation network (**Figure 6A**) comprising 1102 nodes and 3932 links was constructed from cited references. In CiteSpace, clustering quality is evaluated by multiple metrics. Modularity (Q) is used to assess the extent to which the network can be decomposed into relatively independent modules, with values ranging from 0 to 1. Generally, a Q value greater than 0.3 indicates significant clustering. The Silhouette score (S) reflects the internal consistency of each cluster and its separation from other clusters, where values above 0.7 suggest high cluster homogeneity and clear boundaries. As shown in **Figure 6A**, the Q value of the co-citation network is 0.8137, and the S value is 0.9228, indicating strong modularity and clear clustering. Cluster labeling is performed using the log-likelihood ratio (LLR) algorithm, which identifies the most representative terms in each cluster. The higher the LLR value, the more representative the selected label is (18). By using the LLR method, 10 major co-citation clusters (#0–#9) were identified. The largest cluster, #0 “insulin analog”, primarily concentrates on the metabolic and pharmacological mechanisms that link the treatment of diabetes with the risk of pancreatic cancer. The highly co-cited studies in this cluster examine the biological effects of insulin and insulin analogues, as well as their potential roles in pancreatic carcinogenesis. Another core cluster, #1 “cancer risk”, represents the epidemiological foundation of the field. Publications in this cluster focus on population-based cohort and case–control studies to assess the relationship between pancreatic cancer and T2DM incidence rate or prognosis (23). The prominence of this cluster indicates that the relationship between diabetes and pancreatic cancer risk, including the potential role of NOD as an early manifestation, has become a major focus of investigation. Clusters related to therapeutic strategies, including #2 “incretin-based therapy” and #5 “GLP-1 receptor agonist”, reflect research interest in the safety of newer antidiabetic drugs. In addition, several smaller clusters, such as #4 “pancreatic cancer patient”, #6 “antidiabetic drug”, and #8 “diabetes diagnosis”, capture supplementary themes related to clinical characteristics, drug exposure, and diagnostic timing. These clusters complement the major research streams and illustrate the multidimensional nature of studies at the intersection of pancreatic cancer and diabetes. As shown in **Figure 6B**, the arrow originates from a citing cluster and points toward a cited cluster, indicating the direction of co-citation relationships between different research topics. For example, the arrow linking the “diabetes diagnosis” (#8) and “cancer risk” (#1) clusters indicates that research on the identification and classification of diabetes often relies on basic epidemiological research to assess cancer risk.

**Figure 6:**
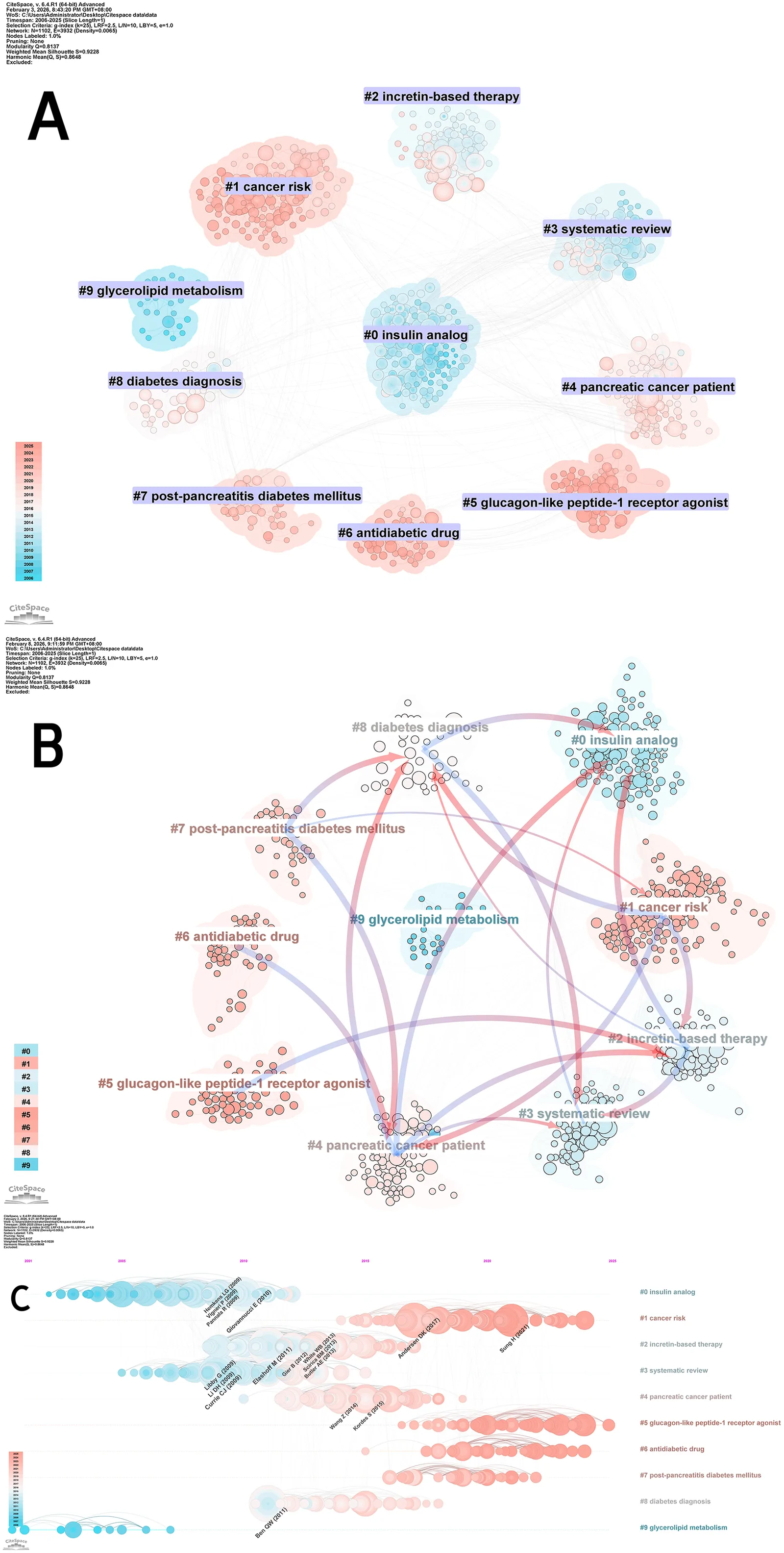
**(A)** Cluster network map of co-cited literature. **(B)** Cluster dependency map of the reference. **(C)** Timeline map of co-cited literature. Each node represents a cited article, and its size is proportional to the frequency of citations. The spatial extent of a cluster directly correlates with the number of cited references it encompasses, reflecting its thematic scale. Cluster labels are numbered in descending order by cluster size and are the most representative topic names assigned to each cluster by the algorithm. A timeline diagram illustrates the chronological evolution of various research themes.

#### Co-cited references timeline analysis

The timeline map illustrates the temporal evolution and research trends of co-cited references across different clusters (18). Based on co-citation clustering, a timeline map comprising the top 10 largest clusters was generated (**Figure 6C**), with earlier studies on the left and more recent studies on the right.

In the early stage, clusters “insulin analog” (#0) and “glycerolipid metabolism” (#9) are predominantly distributed on the left side of the timeline. Research during this period mainly focused on the metabolic characteristics of diabetes, including insulin therapy, lipid metabolism disorders, and their physiological implications. These foundational studies established the metabolic background necessary for later investigations into pancreatic disease and cancer risk. During the intermediate stage, clusters “incretin-based therapy” (#2), “systematic review” (#3), “pancreatic cancer patient” (#4), and “diabetes diagnosis” (#8) became more prominent and concentrated in the middle portion of the timeline. This phase reflects a critical shift in research focus from metabolic mechanisms toward clinical and epidemiological associations. Increasing attention was paid to diabetes as a risk factor for pancreatic cancer and as a potential early manifestation of pancreatic disease, while the emergence of systematic reviews indicates a rapid accumulation of heterogeneous clinical evidence requiring integration. In the recent stage, clusters “glucagon-like peptide-1 receptor agonist” (#5), “antidiabetic drug” (#6), and “post-pancreatitis diabetes mellitus” (#7) are mainly located on the right side of the timeline. Research in this phase emphasizes the long-term safety of antidiabetic medications and the complex bidirectional relationship between diabetes and pancreatic disorders. The diversification of therapeutic strategies and disease subtypes highlights the increasing complexity of the field and the coexistence of multiple research directions.

### Analysis of keywords

#### Keyword co-occurrence analysis

Keyword co-occurrence analysis is conducted to examine the thematic structure of research on pancreatic cancer and T2DM. As shown in **Figure 7A**, the network comprised 563 nodes and 4,500 links, indicating strong interconnections among research topics. The color gradient of the links ranged from blue to pink, representing temporal changes in keyword co-occurrence, with pink links corresponding to more recent research activity. Seven keywords appeared more than 200 times, and 16 appeared more than 100 times. The most frequent keywords are “pancreatic cancer” (555 occurrences), “insulin resistance” (313), “diabetes mellitus” (301), “type 2 diabetes mellitus” (262), “risk” (253), “cancer” (216), and “expression” (205). Terms related to epidemiology, such as “risk” and “cohort study,” are widely distributed in the network. Mechanism-related terms, including “insulin resistance” and “oxidative stress,” are also frequently observed. In addition, therapeutic-related terms such as “metformin” exhibit substantial connectivity in the network, reflecting the coexistence of epidemiological investigations, mechanistic exploration, and treatment research in this field.

**Figure 7:**
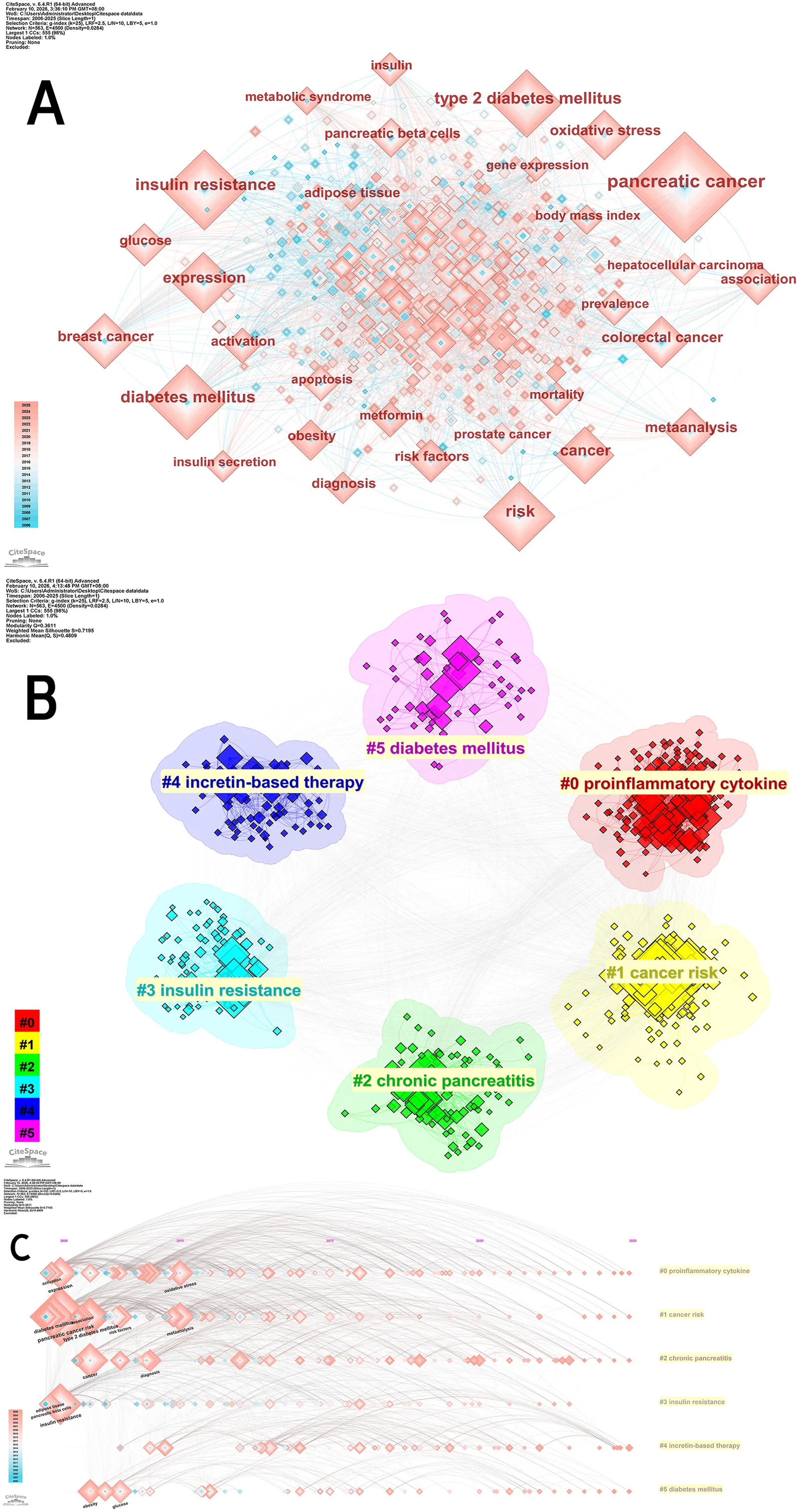
**(A)** Map of keyword co-occurrence. **(B)** Map of keyword clusters. **(C)** Timeline view of the keyword co-occurrence map. Map of keyword co-occurrence illustrating the most frequently used terms and interrelationships. The clustered keyword map displays automatically labeled groups based on thematic similarity, with each cluster representing a major research theme. The timeline view demonstrates the composition of different keywords within each cluster across various periods.

#### Keyword cluster and timeline map analysis

Keyword clustering is performed using the log-likelihood ratio algorithm in CiteSpace. As shown in **Figure 7B**, six major clusters were identified: #0 “proinflammatory cytokine,” #1 “cancer risk,” #2 “chronic pancreatitis,” #3 “insulin resistance,” #4 “incretin-based therapy,” and #5 “diabetes mellitus.” The modularity value is 0.3611, exceeding the 0.3 threshold, indicating significant community structure. The mean silhouette score is 0.7195, suggesting satisfactory internal consistency within clusters. Cluster #0 focuses on inflammation-related terms. Cluster #1 primarily involves epidemiological risk assessment. Cluster #2 is associated with chronic pancreatic inflammatory conditions. Cluster #3 reflects metabolic dysfunction, particularly insulin resistance. Cluster #4 is related to antidiabetic pharmacotherapy. Cluster #5 represents the broader disease background of diabetes mellitus. The clustering results demonstrate that research on pancreatic cancer and T2DM is organized around epidemiological risk, inflammatory processes, metabolic mechanisms, and therapeutic considerations.

**Figure 7C** illustrates that clusters related to metabolic dysfunction (#3 insulin resistance; #5 diabetes mellitus) and epidemiological risk (#1 cancer risk) span the entire study period. Inflammation-associated terms (#0 proinflammatory cytokine) and chronic pancreatic conditions (#2 chronic pancreatitis) remain consistent with the core network. Therapeutic-related keywords (#4 incretin-based therapy) appear in the later segments of the timeline and persist thereafter. The temporal distribution of clustering suggests that research on pancreatic cancer and T2DM is structured around long-standing metabolic and epidemiological themes, with continued integration of inflammatory mechanisms and pharmacological considerations.

#### Keywords with citation bursts

Keyword citation burst detection is performed to identify terms with significantly increased usage during a specific period to reflect shifts in research attention. **Figure 8** shows the top 30 keywords with the strongest citation bursts. The keywords of the early outbreak are mainly related to pancreatic islet biology and experimental models, including “tumor necrosis factor,” “pancreatic islets,” “pancreatic beta cells,” and “NOD mice,” indicating that the initial focus was on β-cell dysfunction and inflammatory mechanisms. From 2010 to 2018, burst terms turned to epidemiological and pharmacological topics, such as “population-based cohort,” “exenatide,” and “reduced risk,” reflecting increased attention to large-scale risk assessment and antidiabetic treatment. The emerging burst keywords include “pancreatic ductal adenocarcinoma,” “mendelian randomization,” “management,” “weight loss,” and “epidemiology,” suggesting growing interest in histology-specific research, causal inference approaches, and early clinical manifestations. The evolution of burst keywords demonstrates a transition from mechanistic studies to population-level analyses, as well as a recent shift towards refined causal and clinical research.

**Figure 8:**
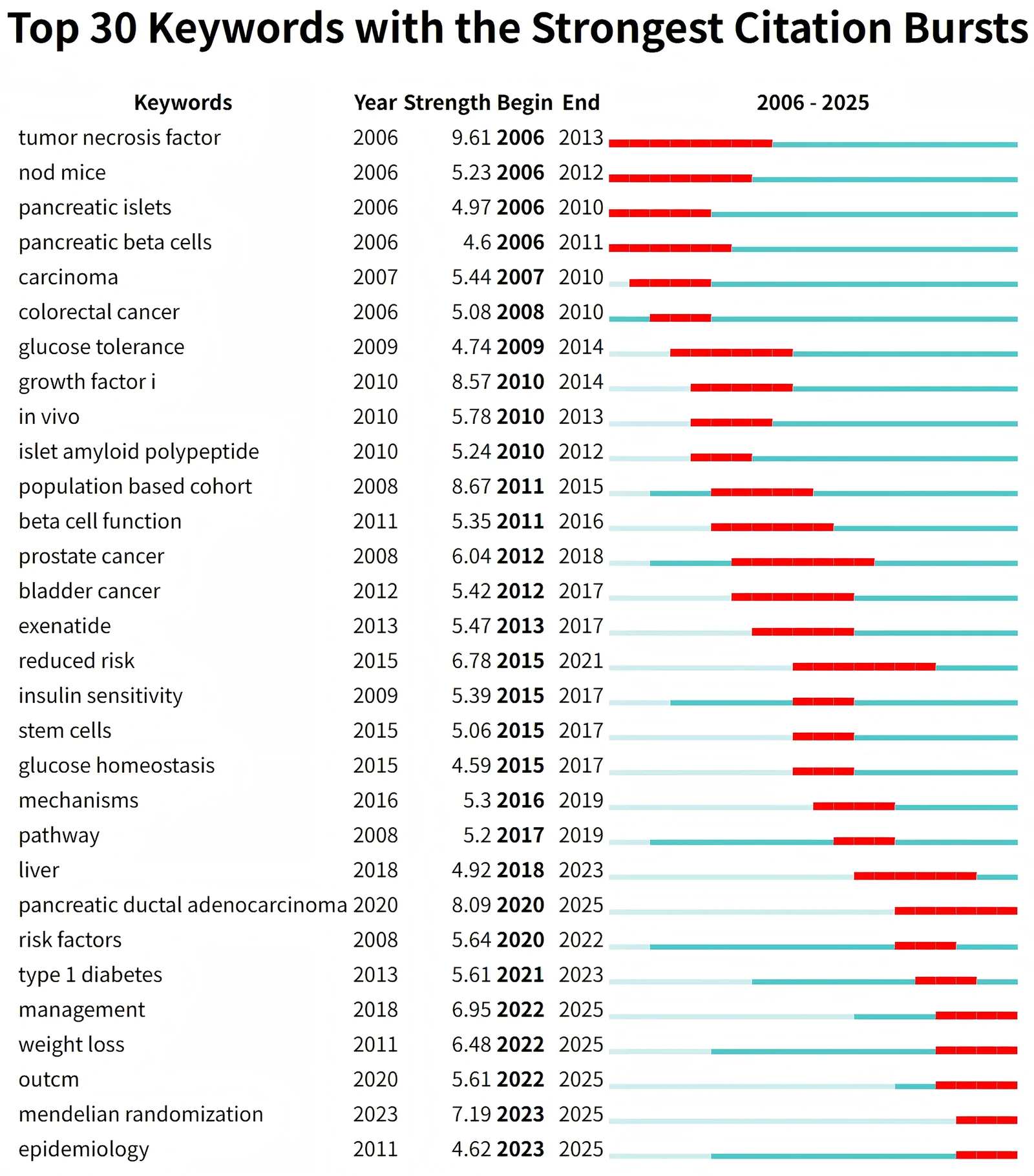
Top 30 keywords with the strongest citation bursts. This image provides the first appearance time of each keyword, the time when the citation burst ended, and their relative intensity. The hot keywords that continue to the present can also be identified through the end time and the red line segment.

### Clinical trial characteristics based on PubMed

To supplement the bibliometric findings from WOSCC, we conducted a supplementary analysis of clinical trial records indexed by PubMed. Using a preset retrieval strategy and limiting the publication type to “clinical trials,” a total of 54 records published between 2006 and 2025 were retrieved. As shown in **Table 4**, the number of clinical trial publications was relatively low before 2014, but significantly increased around 2015, reflecting the increasing interest in translational research and patient-centered research. As shown in **Table 5**, the retrieved studies are mainly published in professional journals of diabetes, oncology and clinical medicine. To provide a more comprehensive characterization of the PubMed dataset, the 54 clinical trial publications were reviewed according to their study design, primary objectives, and major clinical outcomes. Four major categories were identified in **Table 6**. The largest group comprised therapeutic oncology trials, including phase I–III studies evaluating chemotherapy regimens, targeted therapies, mTOR inhibitors, and insulin-like growth factor receptor inhibitors in pancreatic malignancies. These studies primarily assessed progression-free survival, overall survival, tumor response, and treatment-related toxicity. Representative examples include the RADIANT-3 trial, which showed prolonged progression-free survival with everolimus in advanced pancreatic neuroendocrine tumors, as well as multiple phase I/II studies of novel targeted agents. A second category consisted of randomized clinical trials involving patients with T2DM, including TECOS, EXSCEL, LEADER, and ELIXA. These studies mainly focused on cardiovascular outcomes and pancreatic safety. Overall, they did not demonstrate a significant increase in pancreatic cancer incidence associated with the investigated antidiabetic therapies. Several studies focused on early detection and biomarker development. Notable examples included the METAPAC study and the PaCANOD initiative, which investigated metabolite signatures, new-onset diabetes, hyperglycemia, and circulating biomarkers for pancreatic cancer risk stratification and earlier diagnosis. Fewer studies examined endocrine and metabolic outcomes after pancreatic surgery, including islet transplantation and postoperative glycemic management. Collectively, these findings indicate that clinical research has progressively expanded from treatment-oriented trials toward translational approaches involving metabolic risk assessment, early detection, and personalized management strategies.

**Table 4:** Annual publication volume of relevant literature in PubMed in the past decade.

Year	Trials	Year	Trials
2006	2	2016	3
2007	1	2017	6
2008	0	2018	4
2009	4	2019	3
2010	1	2020	2
2011	3	2021	0
2012	3	2022	2
2013	2	2023	0
2014	3	2024	1
2015	13	2025	1

**Table 5:** The top four publications of relevant literature in PubMed.

Journal	Count
*Diabetes Care*	6
*N Engl J Med*	4
*Invest New Drugs*	3
*Clin Cancer Res*	3

**Table 6:** Characteristics of the 54 PubMed-indexed clinical trials.

Research category	Study design	Major endpoints	Main findings
Therapeutic oncology trials	Phase I–III interventional studies	PFS, OS, tumor response, safety	Variable efficacy of chemotherapy and targeted therapies
Diabetes-related trials	Randomized controlled trials	Cardiovascular outcomes, pancreatic safety	No clear increase in pancreatic cancer risk associated with antidiabetic therapies
Early detection and biomarker studies	Prospective screening/diagnostic studies	Diagnostic performance, risk stratification	NOD, hyperglycemia, and biomarkers may aid earlier detection
Surgical and metabolic studies	Prospective clinical studies	Glycemic control, endocrine function	Pancreatic interventions influence metabolic outcomes

## Discussion

### General information

Bibliometric analysis reveals that research on pancreatic cancer and T2DM has gradually shifted from epidemiological association to mechanistic exploration and clinical translation. The steady increase in publications may reflect the growing prevalence of T2DM and the persistent poor prognosis of pancreatic cancer. Growing attention to NOD-related research reflects increasing interest in the temporal and mechanistic relationship between pancreatic cancer and diabetes. The knowledge maps suggest that research in this field primarily focuses on three themes: cancer risk assessment, metabolic inflammation, and treatment safety. Collaborative network analysis indicates that research on the association between pancreatic cancer and T2DM remains relatively dispersed and multi-directional. Consistent with these bibliometric findings, the supplementary analysis of PubMed-indexed clinical trials provided additional insights into therapeutic interventions and metabolic management strategies investigated in clinical research. In particular, recent clinical studies increasingly explored NOD, hyperglycemia, and biomarker-based approaches for pancreatic cancer risk stratification, highlighting the increasing translational interest in moving from observational associations toward potential clinical applications. Bibliometric analyses primarily reflect patterns of scientific attention and knowledge evolution; therefore, the clinical implications discussed below represent contextual interpretations of existing literature rather than direct evidence of clinical efficacy, causality, or implementation.

### Research hotspots

#### Epidemiological risk and bidirectional association

Epidemiological studies have consistently reported an association between T2DM and increased pancreatic cancer risk. The early large-scale cohort analyses from the United States reported that compared with non-diabetic participants, approximately a 1.5-fold higher risk of pancreatic cancer mortality among individuals with diabetes (29). Recent studies suggest that this association is observed across diverse populations and geographic regions (30), suggesting that the relationship between pancreatic cancer and T2DM is globally relevant. In the first few years after diabetes diagnosis, the relative risk is highest (31). A pooled analysis of twelve prospective cohorts from the PanScan consortium demonstrates that the association is most pronounced among individuals with 2–8 years of diabetes duration (32). This study provides evidence that diabetes duration influences pancreatic cancer risk. Although risk estimates tend to attenuate compared with the early post-diagnosis period, longitudinal analyses suggest that pancreatic cancer risk remains elevated across different durations of diabetes (33). These findings suggest that both the onset and duration of diabetes influence pancreatic cancer risk. On the contrary, the prevalence of diabetes in pancreatic cancer patients is very high, especially in the period before cancer diagnosis (34). In a retrospective analysis of 1,556 PDAC cases, 43.2% of patients had diabetes, and approximately 43.5% of these cases were newly diagnosed within two years prior to surgery (35). This indicates a strong temporal clustering of hyperglycemia around the time of cancer detection. In a separate surgical cohort study, 37.9% of PDAC patients with NOD had undiagnosed diabetes until preoperative testing (36), underscoring the frequent under-recognition of early hyperglycemia in the context of occult malignancies. Beyond single-center surgical cohorts, an analysis of large medical databases further confirms this short-term time pattern. Individuals aged ≥50 years with diabetes have been reported to exhibit an elevated risk of pancreatic cancer in the first 12–36 months following diabetes diagnosis (37). Although shared metabolic risk factors and diagnostic bias cannot be entirely excluded, the temporal concentration of risk, together with accumulating mechanistic evidence, has contributed to the recognition of pancreatic cancer–associated diabetes as an emerging clinical concept. The relationship between pancreatic cancer and T2DM has emerged as a complex research area involving metabolic vulnerability and tumor-associated systemic effects, reflecting the increasing interest in mechanistic and translational investigations.

#### Metabolic mechanisms and tumor biology

Chronic hyperinsulinemia is a recognized feature of insulin resistance in T2DM and has been proposed as one mechanism linking diabetes with pancreatic tumor development. Elevated insulin levels activate insulin receptor and IGF-1 signaling, which may enhance mitogenic activity and suppress apoptosis, thereby promoting proliferative capacity in pancreatic ductal epithelial and cancer cells via activation of the PI3K/Akt pathway (38, 39). These findings provide mechanistic evidence supporting a potential relationship between hyperinsulinemia and tumor cell growth. Pharmacologic inhibition of this pathway suppresses cell growth and viability (40, 41). These findings suggest that this pathway may contribute to tumor progression and may represent a potential therapeutic target. Persistent exposure to high insulin levels may promote pancreatic tissue growth, leading to malignant transformation (42). In addition to hyperinsulinemia, chronic low-grade inflammation in T2DM is also a key mechanistic link to pancreatic tumorigenesis (43). Inflammation generates a microenvironment that supports tumor initiation and progression. Experimental models demonstrate that inflammatory signals can accelerate the progression of pancreatic intraepithelial neoplasia (44). Metabolic inflammation is characterized by an increase in pro-inflammatory cytokines like IL-6 and TNF-α (45, 46), activating pro-tumorigenic pathways such as NF-κB and the NFATc1–STAT3 axis, which are implicated in pancreatic tumor initiation (47–49). Clinical research reports indicate that circulating inflammatory biomarkers, including IL-6 and CRP, in pancreatic cancer patients will increase, and the inflammatory burden of metabolic comorbidities such as diabetes is even greater (50, 51). These findings support the notion that systemic inflammation creates a permissive microenvironment that facilitates tumor development. Tumor-secreted factors, including adrenomedullin, impair β-cell function and disrupt glucose homeostasis, and microenvironmental changes such as hypoxia and matrix remodeling further exacerbate insulin resistance, collectively contributing to tumor-associated metabolic disorders (52). Tumor-derived exosomes disrupt glucose regulation by affecting peripheral metabolic tissues, contributing to rapid-onset hyperglycemia (53, 54). These findings support the proposed concept of diabetes as a paraneoplastic phenomenon in pancreatic cancer. PDAC progression can lead to selective β-cell depletion through TGF-β signaling activation (55), further supporting the concept of pancreatic cancer-related diabetes as a paraneoplastic phenomenon. Progressive tumor growth can lead to destruction of pancreatic parenchyma, fibrosis, and remodeling of the pancreatic microenvironment, ultimately impairing endocrine function (56). Pancreatic cancer and diabetes interact through metabolic, inflammatory, and tumor-secretory pathways, suggesting a potentially bidirectional relationship between metabolic dysfunction and tumor progression. Abnormal metabolism promotes tumor growth, and tumor-derived factors worsen blood glucose control (53). The interaction emphasizes the need to consider endocrine and oncological perspectives comprehensively in the pathogenesis of disease in clinical practice.

#### NOD as a potential early detection window

NOD, as a clinically significant interface between pancreatic tumor development and systemic metabolic disorders, represents a clinical interface where tumor-associated metabolic alterations may precede overt cancer diagnosis. In 74–88% of PDAC patients with diabetes, the diabetes diagnosis occurs less than 24 months before the cancer diagnosis, suggesting that NOD may be a paraneoplastic effect of tumors (57). Therefore, positioning NOD not only as a comorbidity but also as a paraneoplastic phenomenon reflecting early tumor-host interactions may offer an opportunity to investigate earlier detection strategies.

Large population-based studies demonstrate a pronounced temporal association between diabetes events and cancer diagnosis, with the highest risk observed in the first three years after the onset of diabetes (58). Individuals with NOD who later develop pancreatic cancer often exhibit distinct clinical characteristics such as ethnic differences, older onset age of diabetes, smoking, and deterioration of glycemic control (30, 59, 60). These observations inform the development of predictive algorithms integrating clinical and metabolic parameters to enrich high-risk populations for targeted surveillance (61, 62). This risk stratification approach represents a potential research direction for enriching high-risk populations and evaluating the feasibility of earlier detection strategies. Meanwhile, the absolute incidence rate of pancreatic cancer in NOD patients is still relatively low (62), and the marked heterogeneity in diabetes pathophysiology complicates the discrimination between tumor-induced diabetes and conventional T2DM in routine clinical settings (63). These challenges limit the immediate clinical implementation of NOD-guided screening strategies while underscoring the urgent need to discover powerful biomarkers, such as circulating proteins and metabolomic features, and to establish effective risk prediction models.

#### Bidirectional therapeutic interactions

The co-cited references timeline analysis indicates increasing research attention toward the therapeutic interactions between pancreatic cancer and T2DM. Metformin, which frequently appears in the keyword co-occurrence analysis, is a commonly used first-line therapy for T2DM. Observational studies have suggested an association between metformin use and improved survival outcomes among pancreatic cancer patients with diabetes (64). However, findings from randomized clinical trials remain inconsistent, and some studies have failed to demonstrate significant survival benefits when metformin is added to standard chemotherapy (65, 66). These discrepancies highlight the uncertainty regarding whether the observed associations reflect potential therapeutic effects or residual confounding. Exogenous insulin therapy has been hypothesized to influence tumor progression through insulin-related mitogenic signaling pathways, although epidemiological findings remain inconclusive (67). Glucagon-like peptide-1 receptor agonist (GLP-1RA) therapies also emerged in the co-cited references timeline analysis. Early reports raised concerns regarding a potential association between incretin-based therapies and pancreatic abnormalities (68). Subsequent population-based studies did not identify a consistent increase in pancreatic cancer risk, highlighting the difficulty of separating potential drug-related effects from the underlying metabolic abnormalities associated with T2DM (69). More recent real-world evidence has further updated this research topic, consistent with the increasing attention to GLP-1RA-related pancreatic safety identified in the bibliometric analysis. A large-scale target trial emulation study using real-world healthcare data reported that GLP-1RA use was not associated with an increased risk of pancreatic cancer compared with other glucose-lowering therapies, although residual confounding and the observational nature of the analysis should still be considered (70). Similarly, a recent large-scale real-world study evaluating GLP-1RA initiation among individuals with T2DM or obesity did not identify a significant increase in pancreatic cancer risk, while emphasizing the importance of continued long-term monitoring (71). These accumulating data illustrate the evolving evaluation of pancreatic safety and potential metabolic effects of antidiabetic therapies, emphasizing the importance of considering both metabolic and oncological outcomes when interpreting emerging evidence.

Therapeutic interventions for pancreatic cancer can substantially influence glucose homeostasis. Surgical resection, especially pancreaticoduodenectomy, is associated with both improvement and deterioration in glycemic control, depending on preoperative metabolic status and the extent of pancreatic tissue removal (72, 73). Clinical studies reported the remission or improvement of diabetes following tumor resection in patients with pancreatic cancer–associated diabetes, supporting the hypothesis that tumor-derived diabetogenic factors may contribute to metabolic dysfunction (74). Postoperative endocrine dysfunction may also occur due to the loss of functional β-cell mass, leading to new-onset or worsening diabetes in some patients (75). These heterogeneous metabolic outcomes underscore the dual role of pancreatic surgery as a potential therapeutic intervention for cancer-associated diabetes and a risk factor for endocrine insufficiency. Beyond surgery, systemic anti-cancer therapies, as well as glucocorticoid-based supportive treatments, may further affect metabolic regulation (76, 77). These findings highlight the importance of individualized glucose monitoring and further investigation into strategies addressing metabolic dysfunction during pancreatic cancer treatment.

### Research frontiers

Recent keyword bursts and co-citation patterns highlight increased research interest in disease diagnosis and clinical treatment methods, reflecting the importance of elucidating the causal biological pathways of the two diseases and developing precise diagnostic and treatment strategies.

### Mechanistic refinement of tumor–metabolic crosstalk

The emerging research focuses more on unraveling the causal relationship between metabolic disorders and pancreatic tumor development through integrated genomic and molecular frameworks. Yuan’s team and Robert C. Torres’s team, respectively, strengthened the evidence for a correlation between increased risk of pancreatic cancer and T2DM, BMI, and fasting insulin levels (78, 79). Srivatsan Raghavan and colleagues studied the dynamic interactions between tumors and abnormal microenvironments and found that basal-like states are associated with EMT, TGF-β signaling, and the cell cycle, which are often activated in T2DM (80). Another study demonstrated that SETD2 deficiency drives metabolic reprogramming in PDAC by establishing a lipid-rich tumor microenvironment, in which lipid-laden cancer-associated fibroblasts supply fatty acids to sustain FAO-OXPHOS and tumor progression (81). Extracellular vesicle-mediated tumor-host communication is an emerging frontier. Lantian Wang and colleagues proposed that exosomal miRNA-let-7b-5p may promote IR in C2C12 myotube cells by targeting RNF20 to activate the STAT3/FOXO1 axis, provided a mechanistic framework for cancer-related metabolic dysfunction, and supported the exploration of circulating factors as candidate biomarkers (82). These advances point toward a systems-level paradigm emphasizing causal inference, metabolic plasticity, and inter-organ signaling as central themes shaping future research trajectories.

### Clinical treatment innovation

Therapeutic innovation is increasingly focused on targeting shared metabolic and signal vulnerability of the interface between pancreatic cancer and T2DM. Caleb Cheng’s team found that inhibiting PIKfyve-driven phosphoinositide metabolism disrupts lipid homeostasis and suppresses tumor growth, highlighting lipid metabolism as a potential therapeutic target (83). The biomarker therapy strategy has also received attention. A glycolysis-related score (GRS) model was developed, and it was found that erlotinib provides the greatest therapeutic benefit for patients with high GRS scores. As a novel prognostic biomarker, the ARNTL2 axis-mediated cellular glycolysis activates the core molecular axis PI3K/AKT signaling pathway, which acts like insulin to exert hypoglycemic effects, making pancreatic cancer cells sensitive to erlotinib therapy and promoting the development of precision oncology (84). The advancement of delivery systems based on extracellular vesicles opened up new avenues for precision therapy. PD-L1 siRNA-loaded CD133-targeting exosomes demonstrated remarkable anticancer efficacy, highlighting their potential as a novel treatment modality (85). These emerging studies demonstrate a shift toward translational risk stratification and precision clinical management strategies that integrate metabolic reprogramming–based therapeutic targets, biomarker-guided drug selection, and innovative delivery platforms to enable personalized and metabolism-oriented interventions at the interface of pancreatic cancer and T2DM.

### Obstructions and prospects

In recent years, the number of studies on pancreatic cancer and T2DM has continued to increase, but the overall research pattern still has some limitations. At the national and institutional levels, research activity remains concentrated in a limited number of high-impact countries and leading institutions, indicating that global collaboration could be further strengthened. The most prolific author contributed only 10 publications, suggesting that research output remains relatively dispersed and that no highly dominant author cluster has yet emerged. Beyond the bibliometric findings, an important diagnostic challenge is distinguishing causal relationships from temporal associations, particularly given the high prevalence of NOD preceding pancreatic cancer diagnosis and the potential for reverse causality in observational studies. Although diabetes is associated with an increased relative risk of pancreatic cancer, the absolute incidence remains relatively low, complicating risk stratification and the implementation of population-based screening strategies (37). Mechanistically, current evidence remains scattered, with most studies focusing on isolated pathways that have not yet been integrated into a network. Prospective biomarker studies further emphasize that circulating metabolites and protein characteristics show promise, but their translation into clinical treatment methods requires validation across different populations (86). Future progress will depend on longitudinal cohort studies, multi-omics integration, the identification of robust biomarkers, and the development of accurate risk-prediction models to clarify the distinct causal relationships between pancreatic cancer and T2DM across patients and to identify high-risk subgroups. Improving treatment precision and integrating systemic metabolic characteristics into comprehensive cancer management represent important directions for future research and clinical translation.

### Limitations

First, this study incorporated a WOSCC-based bibliometric dataset and a supplementary PubMed Clinical Trial dataset, which were analyzed separately and were not merged at any stage of the study. However, other databases such as Scopus, Embase, and Dimensions were not searched. Consequently, some relevant publications and clinical studies may have been missed. Secondly, citation-based analysis reflects the publication and citation model rather than methodological rigor or causality, and, as future pancreatic cancer T2DM research continues to develop, deviations may arise between bibliometric analysis and actual research trends. In addition, visualization tools emphasize network structure and co-occurrence patterns, which limit the ability to evaluate research heterogeneity, confounding, and causal reasoning. Therefore, these findings should be interpreted as a macroscopic overview of research development and knowledge structures rather than definitive mechanistic or causal conclusions.

## Conclusion

This bibliometric analysis outlines the development trajectory of research on the relationship between pancreatic cancer and T2DM. The field has gradually shifted from focusing on epidemiological associations to exploring potential mechanisms and clinical translation. The interaction between tumor development and metabolic disorders has received increasing attention, and NOD has emerged as a key clinical phenomenon and a potential early indicator of pancreatic cancer. The collaborative structure highlights the interdisciplinary nature of this research field. Despite the increasing refinement of research findings and concepts, there are still challenges in elucidating causal relationships and translating mechanistic insights into clinical practice. Future research should emphasize longitudinal design, multi-omics integration, and fine risk stratification to identify high-risk populations, clarify causal pathways, and support early detection and personalized management.

## Data Availability

The original contributions presented in the study are included in the article/supplementary material. Further inquiries can be directed to the corresponding author/s.

## References

[1] Wang S Zheng R Li J Zeng H Li L Chen R . Global, regional, and national lifetime risks of developing and dying from gastrointestinal cancers in 185 countries: a population-based systematic analysis of GLOBOCAN. Lancet Gastroenterol Hepatol. (2024) 9:229–37. doi: 10.1016/s2468-1253(23)00366-7 38185129 PMC10849975

[2] Maisonneuve P Lowenfels AB . Risk factors for pancreatic cancer: a summary review of meta-analytical studies. Int J Epidemiol. (2015) 44:186–98. doi: 10.1093/ije/dyu240 25502106

[3] Clark A Saad MF Nezzer T Uren C Knowler WC Bennett PH . Islet amyloid polypeptide in diabetic and non-diabetic Pima Indians. Diabetologia. (1990) 33:285–9. doi: 10.1007/bf00403322 2198187

[4] Butler AE Janson J Bonner-Weir S Ritzel R Rizza RA Butler PC . Beta-cell deficit and increased beta-cell apoptosis in humans with type 2 diabetes. Diabetes. (2003) 52:102–10. doi: 10.2337/diabetes.52.1.102 12502499

[5] Lee JS Kim SH Jun DW Han JH Jang EC Park JY . Clinical implications of fatty pancreas: correlations between fatty pancreas and metabolic syndrome. World J Gastroenterol. (2009) 15:1869–75. doi: 10.3748/wjg.15.1869 19370785 PMC2670415

[6] Xue A Wu Y Zhu Z Zhang F Kemper KE Zheng Z . Genome-wide association analyses identify 143 risk variants and putative regulatory mechanisms for type 2 diabetes. Nat Commun. (2018) 9:2941. doi: 10.1038/s41467-018-04951-w 30054458 PMC6063971

[7] Hu FB Manson JE Stampfer MJ Colditz G Liu S Solomon CG . Diet, lifestyle, and the risk of type 2 diabetes mellitus in women. N Engl J Med. (2001) 345:790–7. doi: 10.1097/00006254-200203000-00018 11556298

[8] Pang Y Kartsonaki C Guo Y Bragg F Yang L Bian Z . Diabetes, plasma glucose and incidence of pancreatic cancer: A prospective study of 0.5 million Chinese adults and a meta-analysis of 22 cohort studies. Int J Cancer. (2017) 140:1781–8. doi: 10.1002/ijc.30599 28063165 PMC5396360

[9] Hara Y Mizukami H Yamada T Shimoyama S Yamazaki K Sasaki T . Type 2 diabetes alters quiescent pancreatic stellate cells to tumor-prone state. JCI Insight. (2025) 10. doi: 10.1172/jci.insight.187424 40548381 PMC12220947

[10] Zhang AMY Xia YH Lin JSH Chu KH Wang WCK Ruiter TJJ . Hyperinsulinemia acts via acinar insulin receptors to initiate pancreatic cancer by increasing digestive enzyme production and inflammation. Cell Metab. (2023) 35:2119–35:e5. doi: 10.1016/j.cmet.2023.10.003 37913768

[11] Andersen DK Korc M Petersen GM Eibl G Li D Rickels MR . Diabetes, pancreatogenic diabetes, and pancreatic cancer. Diabetes. (2017) 66:1103–10. doi: 10.2337/db16-1477 28507210 PMC5399609

[12] Broadus RN . Toward a definition of “bibliometrics. Scientometrics. (1987) 12:373–9. doi: 10.1007/BF02016680

[13] Ninkov A Frank JR Maggio LA . Bibliometrics: Methods for studying academic publishing. Perspect Med Educ. (2022) 11:173–6. doi: 10.1007/s40037-021-00695-4 34914027 PMC9240160

[14] Chen C . Searching for intellectual turning points: progressive knowledge domain visualization. Proc Natl Acad Sci USA. (2004) 101:5303–10. doi: 10.1073/pnas.0307513100 14724295 PMC387312

[15] Synnestvedt MB Chen C Holmes JH . CiteSpace II: visualization and knowledge discovery in bibliographic databases. AMIA Annu Symp Proc. (2005) 2005:724–8. PMC1560567 16779135

[16] Brandes U . A faster algorithm for betweenness centrality. J Math Sociology. (2001) 25:163–77. doi: 10.1080/0022250x.2001.999024937339054

[17] Freeman LC . Centrality in social networks conceptual clarification. Soc Networks. (1978) 1:215–39. doi: 10.1016/0378-8733(78)90021-7

[18] Chen CM . CiteSpace II: detecting and visualizing emerging trends and transient patterns in scientific literature. J Am Soc For Inf Sci Technol. (2006) 57:359–77. doi: 10.1002/asi.2031742587375

[19] Mathe Z Dupraz P Rinsch C Thorens B Bosco D Zbinden M . Tetracycline-regulated expression of VEGF-A in beta cells induces angiogenesis: improvement of engraftment following transplantation. Cell Transplant. (2006) 15:621–36. doi: 10.3727/000000006783981675 17176614

[20] Elashoff M Matveyenko AV Gier B Elashoff R Butler PC . Pancreatitis, pancreatic, and thyroid cancer with glucagon-like peptide-1-based therapies. Gastroenterology. (2011) 141:150–6. doi: 10.1053/j.gastro.2011.02.018 21334333 PMC4404515

[21] Currie CJ Poole CD Gale EA . The influence of glucose-lowering therapies on cancer risk in type 2 diabetes. Diabetologia. (2009) 52:1766–77. doi: 10.1007/s00125-009-1440-6 19572116

[22] Li D Yeung SC Hassan MM Konopleva M Abbruzzese JL . Antidiabetic therapies affect risk of pancreatic cancer. Gastroenterology. (2009) 137:482–8. doi: 10.1053/j.gastro.2009.04.013 19375425 PMC2735093

[23] Ben Q Xu M Ning X Liu J Hong S Huang W . Diabetes mellitus and risk of pancreatic cancer: a meta-analysis of cohort studies. Eur J Cancer. (2011) 47:1928–37. doi: 10.1016/j.ejca.2011.03.003 21458985

[24] Giovannucci E Harlan DM Archer MC Bergenstal RM Gapstur SM Habel LA . Diabetes and cancer: a consensus report. Diabetes Care. (2010) 33:1674–85. doi: 10.3322/caac.20078 20587728 PMC2890380

[25] Libby G Donnelly LA Donnan PT Alessi DR Morris AD Evans JM . New users of metformin are at low risk of incident cancer: a cohort study among people with type 2 diabetes. Diabetes Care. (2009) 32:1620–5. doi: 10.2337/dc08-2175 19564453 PMC2732153

[26] Sung H Ferlay J Siegel RL Laversanne M Soerjomataram I Jemal A . Global cancer statistics 2020: GLOBOCAN estimates of incidence and mortality worldwide for 36 cancers in 185 countries. CA Cancer J Clin. (2021) 71:209–49. doi: 10.3322/caac.21660 33538338

[27] Butler AE Campbell-Thompson M Gurlo T Dawson DW Atkinson M Butler PC . Marked expansion of exocrine and endocrine pancreas with incretin therapy in humans with increased exocrine pancreas dysplasia and the potential for glucagon-producing neuroendocrine tumors. Diabetes. (2013) 62:2595–604. doi: 10.2337/db12-1686 23524641 PMC3712065

[28] Scirica BM Bhatt DL Braunwald E Steg PG Davidson J Hirshberg B . Saxagliptin and cardiovascular outcomes in patients with type 2 diabetes mellitus. N Engl J Med. (2013) 369:1317–26. doi: 10.1056/nejmoa1307684 23992601

[29] Campbell PT Newton CC Patel AV Jacobs EJ Gapstur SM . Diabetes and cause-specific mortality in a prospective cohort of one million U.S. adults. Diabetes Care. (2012) 35:1835–44. doi: 10.2337/dc12-0002 22699290 PMC3425000

[30] Chari ST Wu B Lopez C Lustigova E Chen Q Van Den Eeden SK . Risk of pancreatic cancer in glycemically defined new-onset diabetes: a prospective cohort study. Gastroenterology. (2026) 170:106–17. doi: 10.1053/j.gastro.2025.06.025 40633624 PMC12403438

[31] Gupta S Vittinghoff E Bertenthal D Corley D Shen H Walter LC . New-onset diabetes and pancreatic cancer. Clin Gastroenterol Hepatol. (2006) 4:1366–72. doi: 10.1016/j.cgh.2006.06.024 16945591

[32] Elena JW Steplowski E Yu K Hartge P Tobias GS Brotzman MJ . Diabetes and risk of pancreatic cancer: a pooled analysis from the pancreatic cancer cohort consortium. Cancer Causes Control. (2013) 24:13–25. doi: 10.1007/s10552-012-0078-8 23112111 PMC3529822

[33] Shen B Li Y Sheng CS Liu L Hou T Xia N . Association between age at diabetes onset or diabetes duration and subsequent risk of pancreatic cancer: Results from a longitudinal cohort and mendelian randomization study. Lancet Reg Health West Pac. (2023) 30:100596. doi: 10.1016/j.lanwpc.2022.100596 36419740 PMC9677075

[34] Chari ST Leibson CL Rabe KG Timmons LJ Ransom J De Andrade M . Pancreatic cancer-associated diabetes mellitus: prevalence and temporal association with diagnosis of cancer. Gastroenterology. (2008) 134:95–101. doi: 10.1053/j.gastro.2007.10.040 18061176 PMC2271041

[35] Kanbour S Yenokyan G Abusamaan M Laheru D Alam A El Asmar ML . Association of long-term, new-onset, and postsurgical diabetes with survival in patients with resectable pancreatic cancer: a retrospective cohort study. Pancreas. (2023) 52:e309–14. doi: 10.1097/mpa.0000000000002257 37890159 PMC11913223

[36] Stott M Stefanova I Oldfield L Evans A Birch-Ford J Rao R . Prevalence of new-onset diabetes in patients undergoing pancreatic surgery and the association of glucose dysregulation with complications in pancreatic cancer. Ann Surg Open. (2025) 6:e584. doi: 10.1097/as9.0000000000000584 40557340 PMC12185084

[37] Jensen MH Cichosz SL Hejlesen O Henriksen SD Drewes AM Olesen SS . Risk of pancreatic cancer in people with new-onset diabetes: a Danish nationwide population-based cohort study. Pancreatology. (2023) 23:642–9. doi: 10.1016/j.pan.2023.07.001 37422338

[38] Ma J Sawai H Matsuo Y Ochi N Yasuda A Takahashi H . IGF-1 mediates PTEN suppression and enhances cell invasion and proliferation via activation of the IGF-1/PI3K/Akt signaling pathway in pancreatic cancer cells. J Surg Res. (2010) 160:90–101. doi: 10.1016/j.jss.2008.08.016 19560785

[39] Zhang AMY Magrill J De Winter TJJ Hu X Skovso S Schaeffer DF . Endogenous hyperinsulinemia contributes to pancreatic cancer development. Cell Metab. (2019) 30:403–4. doi: 10.1016/j.cmet.2019.07.003 31378465

[40] Liu S Wang XJ Liu Y Cui YF . PI3K/AKT/mTOR signaling is involved in (-)-epigallocatechin-3-gallate-induced apoptosis of human pancreatic carcinoma cells. Am J Chin Med. (2013) 41:629–42. doi: 10.1142/s0192415x13500444 23711146

[41] Xie P Tan SY Li HF Tang HD Zhou JH . Transcriptome data-based status of PI3K/AKT/mTOR pathway indicates heterogeneity and immune modulation in patients with pancreatic ductal adenocarcinoma. J Gene Med. (2024) 26:e3570. doi: 10.1002/jgm.3570 37482968

[42] Zhang AMY Chu KH Daly BF Ruiter T Dou Y Yang JCC . Effects of hyperinsulinemia on pancreatic cancer development and the immune microenvironment revealed through single-cell transcriptomics. Cancer Metab. (2022) 10:5. doi: 10.1186/s40170-022-00282-z 35189981 PMC8862319

[43] Babic A Schnure N Neupane NP Zaman MM Rifai N Welch MW . Plasma inflammatory cytokines and survival of pancreatic cancer patients. Clin Transl Gastroenterol. (2018) 9:145. doi: 10.1038/s41424-018-0008-5 29691365 PMC5915593

[44] Lesina M Kurkowski MU Ludes K Rose-John S Treiber M Kloppel G . Stat3/Socs3 activation by IL-6 transsignaling promotes progression of pancreatic intraepithelial neoplasia and development of pancreatic cancer. Cancer Cell. (2011) 19:456–69. doi: 10.1016/j.ccr.2011.03.009 21481788

[45] Kern PA Ranganathan S Li C Wood L Ranganathan G . Adipose tissue tumor necrosis factor and interleukin-6 expression in human obesity and insulin resistance. Am J Physiol Endocrinol Metab. (2001) 280:E745–51. doi: 10.1152/ajpendo.2001.280.5.e745 11287357

[46] Pang Y Lv J Wu T Yu C Guo Y Chen Y . Associations of diabetes, circulating protein biomarkers, and risk of pancreatic cancer. Br J Cancer. (2024) 130:504–10. doi: 10.1038/s41416-023-02533-2 38129526 PMC10844301

[47] Baumgart S Chen NM Siveke JT Konig A Zhang JS Singh SK . Inflammation-induced NFATc1-STAT3 transcription complex promotes pancreatic cancer initiation by KrasG12D. Cancer Discov. (2014) 4:688–701. doi: 10.1158/2159-8290.cd-13-0593 24694735 PMC4069603

[48] Daniluk J Liu Y Deng D Chu J Huang H Gaiser S . An NF-kappaB pathway-mediated positive feedback loop amplifies Ras activity to pathological levels in mice. J Clin Invest. (2012) 122:1519–28. doi: 10.1172/jci59743 22406536 PMC3314459

[49] Rajurkar M De Jesus-Monge WE Driscoll DR Appleman VA Huang H Cotton JL . The activity of Gli transcription factors is essential for Kras-induced pancreatic tumorigenesis. Proc Natl Acad Sci USA. (2012) 109:E1038–47. doi: 10.1073/pnas.1114168109 22493246 PMC3340052

[50] Kolb H Mandrup-Poulsen T . The global diabetes epidemic as a consequence of lifestyle-induced low-grade inflammation. Diabetologia. (2010) 53:10–20. doi: 10.14341/2071-8713-5089 19890624

[51] Singh N Gupta S Rashid S Saraya A . Association of inflammatory markers with the disease & mutation status in pancreatic cancer. Indian J Med Res. (2022) 155:49–55. doi: 10.4103/ijmr.ijmr_2238_18 35859428 PMC9552386

[52] Aggarwal G Ramachandran V Javeed N Arumugam T Dutta S Klee GG . Adrenomedullin is up-regulated in patients with pancreatic cancer and causes insulin resistance in beta cells and mice. Gastroenterology. (2012) 143:1510–7:e1. doi: 10.1007/978-1-4419-1623-5_62 PMC3787599 22960655

[53] Javeed N Sagar G Dutta SK Smyrk TC Lau JS Bhattacharya S . Pancreatic cancer-derived exosomes cause paraneoplastic beta-cell dysfunction. Clin Cancer Res. (2015) 21:1722–33. doi: 10.1158/1078-0432.CCR-14-2022 PMC4383684 25355928

[54] Wang L Zhang B Zheng W Kang M Chen Q Qin W . Exosomes derived from pancreatic cancer cells induce insulin resistance in C2C12 myotube cells through the PI3K/Akt/FoxO1 pathway. Sci Rep. (2017) 7:5384. doi: 10.1038/s41598-017-05541-4 28710412 PMC5511275

[55] Parajuli P Nguyen TL Prunier C Razzaque MS Xu K Atfi A . Pancreatic cancer triggers diabetes through TGF-beta-mediated selective depletion of islet beta-cells. Life Sci Alliance. (2020) 3. doi: 10.26508/lsa.201900573 PMC7211975 32371554

[56] Hu K Zhao X Zhang N Ma J Zhang R Lu Z . Effect of tumor microenvironment in pancreatic cancer on the loss of beta-cell mass: implications for type 3c diabetes. J Gastroenterol. (2025) 60:512–25. doi: 10.1007/s00535-024-02204-w 39760782

[57] Pannala R Leirness JB Bamlet WR Basu A Petersen GM Chari ST . Prevalence and clinical profile of pancreatic cancer-associated diabetes mellitus. Gastroenterology. (2008) 134:981–7. doi: 10.1053/j.gastro.2008.01.039 18395079 PMC2323514

[58] Setiawan VW Stram DO Porcel J Chari ST Maskarinec G Le Marchand L . Pancreatic cancer following incident diabetes in African Americans and Latinos: The Multiethnic Cohort. J Natl Cancer Inst. (2019) 111:27–33. doi: 10.1093/jnci/djy090 29917105 PMC6335114

[59] Chari ST Leibson CL Rabe KG Ransom J De Andrade M Petersen GM . Probability of pancreatic cancer following diabetes: a population-based study. Gastroenterology. (2005) 129:504–11. doi: 10.1016/j.gastro.2005.05.007 16083707 PMC2377196

[60] Grote VA Rohrmann S Nieters A Dossus L Tjonneland A Halkjaer J . Diabetes mellitus, glycated haemoglobin and C-peptide levels in relation to pancreatic cancer risk: a study within the European Prospective Investigation into Cancer and Nutrition (EPIC) cohort. Diabetologia. (2011) 54:3037–46. doi: 10.1007/s00125-011-2316-0 21953276

[61] Boursi B Finkelman B Giantonio BJ Haynes K Rustgi AK Rhim AD . A clinical prediction model to assess risk for pancreatic cancer among patients with new-onset diabetes. Gastroenterology. (2017) 152:840–50:e3. doi: 10.1053/j.gastro.2016.11.046 27923728 PMC5337138

[62] Sharma A Kandlakunta H Nagpal SJS Feng Z Hoos W Petersen GM . Model to determine risk of pancreatic cancer in patients with new-onset diabetes. Gastroenterology. (2018) 155:730–9:e3. doi: 10.1053/j.gastro.2018.05.023 29775599 PMC6120785

[63] Zhang T Hua X Mohindroo C Wang X Dutta D Liu J . Different diabetes types and pancreatic ductal adenocarcinoma: a mendelian randomization and pathway/gene-set analysis. J Natl Cancer Inst. (2026) 118(3):437–47. doi: 10.1093/jnci/djaf308 41206949 PMC13005432

[64] Dulskas A Patasius A Linkeviciute-Ulinskiene D Zabuliene L Smailyte G . Cohort study of antihyperglycemic medication and pancreatic cancer patients survival. Int J Environ Res Public Health. (2020) 17. doi: 10.3390/ijerph17176016 32824907 PMC7503289

[65] Cerullo M Gani F Chen SY Canner J Pawlik TM . Metformin use is associated with improved survival in patients undergoing resection for pancreatic cancer. J Gastrointest Surg. (2016) 20:1572–80. doi: 10.1007/s11605-016-3173-4 27255657

[66] Kordes S Pollak MN Zwinderman AH Mathot RA Weterman MJ Beeker A . Metformin in patients with advanced pancreatic cancer: a double-blind, randomised, placebo-controlled phase 2 trial. Lancet Oncol. (2015) 16:839–47. doi: 10.1016/s1470-2045(15)00027-3 26067687

[67] Chan MT Lim GE Skovso S Yang YH Albrecht T Alejandro EU . Effects of insulin on human pancreatic cancer progression modeled *in vitro*. BMC Cancer. (2014) 14:814. doi: 10.1186/1471-2407-14-814 25373319 PMC4233074

[68] Singh S Chang HY Richards TM Weiner JP Clark JM Segal JB . Glucagonlike peptide 1-based therapies and risk of hospitalization for acute pancreatitis in type 2 diabetes mellitus: a population-based matched case-control study. JAMA Intern Med. (2013) 173:534–9. doi: 10.1001/jamainternmed.2013.2720 23440284

[69] Azoulay L Filion KB Platt RW Dahl M Dormuth CR Clemens KK . Incretin based drugs and the risk of pancreatic cancer: international multicentre cohort study. BMJ. (2016) 352:i581. doi: 10.1136/bmj.i581 26888382 PMC4772785

[70] Wang L Wang Q Li L Kaelber DC Xu R . Glucagon-like peptide-1 receptor agonists and pancreatic cancer risk: target trial emulation using real-world data. J Natl Cancer Inst. (2025) 117:476–85. doi: 10.1093/jnci/djae260 39418202 PMC11884861

[71] Tartaglia E Bucci T Rossi M Rigutini AG Askarinejad A Alam U . Risk of all-cause death and pancreatic events following GLP-1 RA initiation in people with obesity or type 2 diabetes: observations from a federated research network. Cardiovasc Diabetol. (2025) 24:438. doi: 10.1186/s12933-025-02986-0 41257737 PMC12628983

[72] Maxwell DW Jajja MR Tariq M Mahmooth Z Galindo RJ Sweeney JF . Development of diabetes after pancreaticoduodenectomy: Results of a 10-year series using prospective endocrine evaluation. J Am Coll Surg. (2019) 228:400–12:e2. doi: 10.1016/j.jamcollsurg.2018.12.042 30690075

[73] Niwano F Babaya N Hiromine Y Matsumoto I Kamei K Noso S . Glucose metabolism after pancreatectomy: Opposite extremes between pancreaticoduodenectomy and distal pancreatectomy. J Clin Endocrinol Metab. (2021) 106:e2203–14. doi: 10.1210/clinem/dgab036 33484558 PMC8063252

[74] He XY Li JF Yao WY Yuan YZ . Resolution of new-onset diabetes after radical pancreatic resection predicts long-term survival in patients with pancreatic ductal cell adenocarcinoma. Ann Surg Oncol. (2013) 20:3809–16. doi: 10.1245/s10434-013-3095-2 23943021

[75] Imamura S Niwano F Babaya N Hiromine Y Matsumoto I Kamei K . High incidence of diabetes mellitus after distal pancreatectomy and its predictors: a long-term follow-up study. J Clin Endocrinol Metab. (2024) 109:619–30. doi: 10.1210/clinem/dgad634 37889837 PMC10876401

[76] Eriksen M Jensen DH Tribler S Holst JJ Madsbad S Krarup T . Reduction of insulinotropic properties of GLP-1 and GIP after glucocorticoid-induced insulin resistance. Diabetologia. (2015) 58:920–8. doi: 10.1007/s00125-015-3522-y 25748606

[77] Yao JC Shah MH Ito T Bohas CL Wolin EM Van Cutsem E . Everolimus for advanced pancreatic neuroendocrine tumors. N Engl J Med. (2011) 364:514–23. doi: 10.1056/nejmoa1009290 21306238 PMC4208619

[78] Carreras-Torres R Johansson M Gaborieau V Haycock PC Wade KH Relton CL . The role of obesity, type 2 diabetes, and metabolic factors in pancreatic cancer: a Mendelian randomization study. J Natl Cancer Inst. (2017) 109. doi: 10.1093/jnci/djx012 28954281 PMC5721813

[79] Yuan S Kar S Carter P Vithayathil M Mason AM Burgess S . Is type 2 diabetes causally associated with cancer risk? Evidence from a two-sample mendelian randomization study. Diabetes. (2020) 69:1588–96. doi: 10.2337/db20-0084 32349989 PMC7306131

[80] Raghavan S Winter PS Navia AW Williams HL Denadel A Lowder KE . Microenvironment drives cell state, plasticity, and drug response in pancreatic cancer. Cell. (2021) 184:6119–37:e26. doi: 10.1016/j.cell.2021.11.017 34890551 PMC8822455

[81] Niu N Shen X Wang Z Chen Y Weng Y Yu F . Tumor cell-intrinsic epigenetic dysregulation shapes cancer-associated fibroblasts heterogeneity to metabolically support pancreatic cancer. Cancer Cell. (2024) 42:869–84:e9. doi: 10.1016/j.ccell.2024.03.005 38579725

[82] Wang L Li X Wu J Tang Q . Pancreatic cancer-derived exosomal miR-Let-7b-5p stimulates insulin resistance in skeletal muscle cells through RNF20/STAT3/FOXO1 axis regulation. Diabetes Metab Syndr Obes. (2023) 16:3133–45. doi: 10.2147/dmso.s430443 37842335 PMC10573399

[83] Cheng C Hu J Mannan R He T Bhattacharyya R Magnuson B . Targeting PIKfyve-driven lipid metabolism in pancreatic cancer. Nature. (2025) 642:776–84. doi: 10.1038/s41586-025-08917-z 40269157 PMC12176661

[84] Ge W Wang Y Quan M Mao T Bischof EY Xu H . Activation of the PI3K/AKT signaling pathway by ARNTL2 enhances cellular glycolysis and sensitizes pancreatic adenocarcinoma to erlotinib. Mol Cancer. (2024) 23:48. doi: 10.1186/s12943-024-01965-5 38459558 PMC10921723

[85] Yoon YC Lee D Park JH Kim OH Choi HJ Kim SJ . Enhancing pancreatic cancer therapy with targeted CD133-exosome delivery of PD-L1 siRNA: a preclinical investigation. Pancreas. (2025) 54:e210–20. doi: 10.1097/mpa.0000000000002419 39590886 PMC11882183

[86] Cohen JD Li L Wang Y Thoburn C Afsari B Danilova L . Detection and localization of surgically resectable cancers with a multi-analyte blood test. Science. (2018) 359:926–30. doi: 10.1126/science.aar3247 29348365 PMC6080308

